# Supplementary material for: A Likelihood Approach for Real-Time Calibration of Stochastic Compartmental Epidemic Models
Source: PLoS Comput Biol. 2017 Jan 17;13(1):e1005257. doi: 10.1371/journal.pcbi.1005257 (PMC5240920; doi:10.1371/journal.pcbi.1005257)
Supplement: S1 File — (TAR.GZ) [file pcbi.1005257.s014.tar.gz › HSPH_Online-SI-Revision/PF08/n10-extreme/n10-extreme_PF2_table0.pdf]

| | | | | | | | | | | | | | | | | | | | | | | | | | | | | | | |
|---|---|---|---|---|---|---|---|---|---|---|---|---|---|---|---|---|---|---|---|---|---|---|---|---|---|---|---|---|---|---|
| 1 | newly 3 weeks spec | 724. | 867.45 | {820., 917.} | 19.8384 | 0.0780001 | newly 3 weeks cumul | 3019. | 3073.55 | {2960., 3218.} | 3.20934 | 0.0138212 | newly 5 weeks spec | 306. | 543.89 | {507., 582.} | 77.7418 | 0.249044 | newly 5 weeks cumul | 3848. | 4311.77 | {4181., 4508.} | 12.2477 | 0.0499528 | newly 58 weeks cumul | 4437. | 6016.35 | {5834., 6327.} | 35.595 | 0.131852 |
| 2 | newly 3 weeks spec | 551. | 542.92 | {486., 588.} | 5.79673 | 0.025745 | newly 3 weeks cumul | 1831. | 1712.71 | {1609., 1827.} | 6.82523 | 0.0311859 | newly 5 weeks spec | 397. | 436.16 | {389., 477.} | 11.2997 | 0.0460079 | newly 5 weeks cumul | 2702. | 2644.76 | {2482., 2833.} | 4.42413 | 0.0197175 | newly 54 weeks cumul | 4210. | 4880.16 | {4497., 5267.} | 16.1425 | 0.0642085 |
| 3 | newly 3 weeks spec | 586. | 774.24 | {740., 814.} | 32.1229 | 0.120677 | newly 3 weeks cumul | 2929. | 2946.2 | {2888., 3012.} | 1.39638 | 0.00600139 | newly 5 weeks spec | 256. | 439.79 | {408., 466.} | 71.793 | 0.234414 | newly 5 weeks cumul | 3549. | 3979.19 | {3902., 4049.} | 12.1214 | 0.0496268 | newly 60 weeks cumul | 3885. | 5161.96 | {5079., 5245.} | 32.869 | 0.12337 |
| 4 | newly 3 weeks spec | 581. | 561.37 | {513., 602.} | 5.30981 | 0.0239503 | newly 3 weeks cumul | 2166. | 1901.14 | {1809., 1985.} | 12.2281 | 0.0569497 | newly 5 weeks spec | 320. | 408.58 | {357., 450.} | 27.7188 | 0.104613 | newly 5 weeks cumul | 2954. | 2791.11 | {2624., 2937.} | 5.67942 | 0.0257869 | newly 54 weeks cumul | 3942. | 4496.09 | {3994., 4863.} | 14.7369 | 0.0588495 |
| 5 | newly 3 weeks spec | 669. | 740.49 | {661., 806.} | 11.3528 | 0.0459781 | newly 3 weeks cumul | 2580. | 2643.72 | {2444., 2813.} | 4.90775 | 0.0209226 | newly 5 weeks spec | 386. | 469.16 | {418., 519.} | 22.0104 | 0.0852791 | newly 5 weeks cumul | 3457. | 3707.86 | {3408., 3941.} | 8.04975 | 0.0332885 | newly 57 weeks cumul | 4587. | 5224.61 | {4760., 5644.} | 14.1812 | 0.0569426 |
| 6 | newly 3 weeks spec | 808. | 820.29 | {745., 885.} | 5.32054 | 0.0228836 | newly 3 weeks cumul | 2954. | 2826.28 | {2623., 2972.} | 4.71429 | 0.021364 | newly 5 weeks spec | 362. | 539.04 | {479., 590.} | 48.9061 | 0.171631 | newly 5 weeks cumul | 3891. | 4039.12 | {3697., 4268.} | 5.6402 | 0.0238737 | newly 58 weeks cumul | 4944. | 5855.93 | {5326., 6263.} | 18.4464 | 0.0727873 |
| 7 | newly 3 weeks spec | 755. | 851.49 | {786., 930.} | 12.8252 | 0.0516086 | newly 3 weeks cumul | 2728. | 2873.98 | {2696., 2996.} | 5.90469 | 0.0246832 | newly 5 weeks spec | 406. | 560.78 | {509., 612.} | 38.1232 | 0.139043 | newly 5 weeks cumul | 3682. | 4136.6 | {3896., 4333.} | 12.3466 | 0.0501253 | newly 59 weeks cumul | 4694. | 5972.95 | {5471., 6327.} | 27.2465 | 0.103914 |
| 8 | newly 3 weeks spec | 735. | 691.76 | {646., 737.} | 6.42721 | 0.0292213 | newly 3 weeks cumul | 2632. | 2321.04 | {2234., 2410.} | 11.8146 | 0.0548381 | newly 5 weeks spec | 475. | 490.59 | {453., 529.} | 5.68632 | 0.0240372 | newly 5 weeks cumul | 3719. | 3403.55 | {3278., 3518.} | 8.48212 | 0.0387187 | newly 56 weeks cumul | 5155. | 5298.45 | {5007., 5501.} | 4.0677 | 0.017326 |
| 9 | newly 3 weeks spec | 675. | 703.6 | {650., 747.} | 5.76296 | 0.0242986 | newly 3 weeks cumul | 2453. | 2398.17 | {2262., 2505.} | 3.36445 | 0.0150058 | newly 5 weeks spec | 427. | 482.24 | {443., 519.} | 13.1429 | 0.0529139 | newly 5 weeks cumul | 3435. | 3465.85 | {3301., 3575.} | 2.9313 | 0.0127209 | newly 56 weeks cumul | 4640. | 5236.64 | {5013., 5465.} | 12.9384 | 0.0525626 |
| 10 | newly 3 weeks spec | 697. | 687.8 | {617., 742.} | 5.2396 | 0.0232751 | newly 3 weeks cumul | 2508. | 2242.4 | {2009., 2357.} | 10.5901 | 0.0492973 | newly 5 weeks spec | 467. | 501.91 | {450., 551.} | 9.0257 | 0.0370356 | newly 5 weeks cumul | 3582. | 3341.25 | {3018., 3511.} | 6.74009 | 0.0309134 | newly 56 weeks cumul | 5280. | 5332.54 | {5028., 5665.} | 4.05568 | 0.0174345 |
| 11 | newly 3 weeks spec | 649. | 523.65 | {495., 560.} | 19.3143 | 0.0937835 | newly 3 weeks cumul | 2299. | 1689.25 | {1629., 1740.} | 26.5224 | 0.134055 | newly 5 weeks spec | 447. | 421.14 | {390., 447.} | 6.42058 | 0.0292434 | newly 5 weeks cumul | 3330. | 2584.78 | {2512., 2671.} | 22.379 | 0.110183 | newly 51 weeks cumul | 4840. | 4755.42 | {4594., 4920.} | 2.53554 | 0.0112248 |
| 12 | newly 3 weeks spec | 799. | 689.59 | {644., 730.} | 13.6934 | 0.064513 | newly 3 weeks cumul | 2755. | 2225.24 | {2106., 2329.} | 19.229 | 0.0931338 | newly 5 weeks spec | 494. | 517.01 | {478., 557.} | 6.28138 | 0.0261578 | newly 5 weeks cumul | 3861. | 3347.03 | {3218., 3462.} | 13.3118 | 0.0622837 | newly 56 weeks cumul | 5044. | 5447.37 | {5134., 5807.} | 8.18418 | 0.0337459 |
| 13 | newly 3 weeks spec | 580. | 492.5 | {446., 531.} | 15.0862 | 0.0718879 | newly 3 weeks cumul | 2137. | 1641.59 | {1580., 1710.} | 23.1825 | 0.114759 | newly 5 weeks spec | 319. | 370.26 | {338., 402.} | 16.1693 | 0.0639688 | newly 5 weeks cumul | 2908. | 2442.01 | {2364., 2544.} | 16.0244 | 0.07605 | newly 52 weeks cumul | 3866. | 4175.68 | {3915., 4562.} | 8.12209 | 0.0332101 |
| 14 | newly 3 weeks spec | 614. | 704.09 | {600., 777.} | 15.9397 | 0.063252 | newly 3 weeks cumul | 2380. | 2485.12 | {2158., 2678.} | 8.15042 | 0.034543 | newly 5 weeks spec | 361. | 456.59 | {372., 519.} | 27.3102 | 0.102703 | newly 5 weeks cumul | 3257. | 3515.4 | {3027., 3787.} | 10.7326 | 0.0444493 | newly 57 weeks cumul | 4210. | 5072.4 | {4231., 5556.} | 21.4945 | 0.0833748 |
| 15 | newly 3 weeks spec | 533. | 577.45 | {537., 615.} | 9.12383 | 0.0375875 | newly 3 weeks cumul | 2029. | 1926.74 | {1826., 2016.} | 5.18581 | 0.0235401 | newly 5 weeks spec | 261. | 428.22 | {390., 467.} | 64.069 | 0.213817 | newly 5 weeks cumul | 2645. | 2860.2 | {2730., 2978.} | 8.6276 | 0.0358013 | newly 54 weeks cumul | 3346. | 4734.87 | {4332., 4989.} | 41.5084 | 0.150073 |
| 16 | newly 3 weeks spec | 754. | 856.01 | {807., 902.} | 13.6406 | 0.0550998 | newly 3 weeks cumul | 2774. | 2946.02 | {2778., 3060.} | 6.76424 | 0.028331 | newly 5 weeks spec | 425. | 556.07 | {510., 605.} | 30.84 | 0.115781 | newly 5 weeks cumul | 3788. | 4202.44 | {4008., 4327.} | 11.0639 | 0.0454135 | newly 57 weeks cumul | 4918. | 6063.21 | {5854., 6358.} | 23.2861 | 0.0906161 |
| 17 | newly 3 weeks spec | 715. | 831.54 | {763., 896.} | 16.5315 | 0.0657461 | newly 3 weeks cumul | 2771. | 2928.16 | {2714., 3103.} | 6.66907 | 0.0278858 | newly 5 weeks spec | 457. | 527.21 | {486., 575.} | 16.0766 | 0.0638866 | newly 5 weeks cumul | 3767. | 4125.22 | {3839., 4336.} | 9.94107 | 0.0408693 | newly 59 weeks cumul | 4791. | 5807.34 | {5253., 6190.} | 21.3016 | 0.0831236 |
| 18 | newly 3 weeks spec | 721. | 753.89 | {708., 803.} | 5.56033 | 0.0232664 | newly 3 weeks cumul | 2736. | 2694.82 | {2584., 2818.} | 3.04825 | 0.0134585 | newly 5 weeks spec | 402. | 477.09 | {434., 519.} | 18.6791 | 0.0733665 | newly 5 weeks cumul | 3695. | 3776.66 | {3633., 3948.} | 3.33802 | 0.0142078 | newly 57 weeks cumul | 4724. | 5321. | {5061., 5631.} | 12.6461 | 0.0513187 |
| 19 | newly 3 weeks spec | 716. | 927.15 | {860., 1003.} | 29.4902 | 0.111404 | newly 3 weeks cumul | 3184. | 3208.99 | {3072., 3325.} | 2.77858 | 0.0120815 | newly 5 weeks spec | 379. | 587.02 | {522., 664.} | 54.8865 | 0.188187 | newly 5 weeks cumul | 4076. | 4544.1 | {4342., 4794.} | 11.7709 | 0.0480742 | newly 59 weeks cumul | 4764. | 6379.04 | {5918., 6942.} | 33.9009 | 0.125931 |
| 20 | newly 3 weeks spec | 765. | 719.66 | {670., 768.} | 6.25098 | 0.0284423 | newly 3 weeks cumul | 2647. | 2423.12 | {2289., 2537.} | 8.45788 | 0.0387146 | newly 5 weeks spec | 468. | 505.38 | {466., 541.} | 8.48291 | 0.0348191 | newly 5 weeks cumul | 3727. | 3543.48 | {3409., 3686.} | 4.95144 | 0.0222585 | newly 56 weeks cumul | 5189. | 5452.96 | {5225., 5630.} | 5.29852 | 0.0222538 |
| 21 | newly 3 weeks spec | 673. | 550.76 | {515., 589.} | 18.1634 | 0.0876511 | newly 3 weeks cumul | 2256. | 1792.21 | {1720., 1883.} | 20.5581 | 0.100187 | newly 5 weeks spec | 405. | 425.55 | {384., 460.} | 7.05926 | 0.0295107 | newly 5 weeks cumul | 3166. | 2708.35 | {2586., 2826.} | 14.4551 | 0.0680845 | newly 53 weeks cumul | 4383. | 4711.05 | {4400., 4945.} | 7.71869 | 0.0319798 |
| 22 | newly 3 weeks spec | 723. | 840.84 | {809., 875.} | 16.2988 | 0.0653505 | newly 3 weeks cumul | 3471. | 3095.89 | {3026., 3160.} | 10.807 | 0.0497271 | newly 5 weeks spec | 278. | 478.45 | {435., 520.} | 72.1043 | 0.234749 | newly 5 weeks cumul | 4204. | 4218.67 | {4151., 4286.} | 1.07826 | 0.00465379 | newly 60 weeks cumul | 4711. | 5465.2 | {5308., 5672.} | 16.0093 | 0.0642624 |
| 23 | newly 3 weeks spec | 587. | 582.75 | {512., 652.} | 7.35434 | 0.0324376 | newly 3 weeks cumul | 1900. | 1905.45 | {1690., 2123.} | 7.95421 | 0.0346237 | newly 5 weeks spec | 330. | 442.14 | {382., 506.} | 34.0545 | 0.124998 | newly 5 weeks cumul | 2657. | 2864.59 | {2579., 3167.} | 9.49454 | 0.0387934 | newly 55 weeks cumul | 3467. | 4871.32 | {4383., 5433.} | 40.7707 | 0.147206 |
| 24 | newly 3 weeks spec | 579. | 535.67 | {496., 563.} | 7.67358 | 0.0351721 | newly 3 weeks cumul | 2054. | 1825.17 | {1731., 1898.} | 11.1407 | 0.0515965 | newly 5 weeks spec | 382. | 385.99 | {346., 417.} | 5.99215 | 0.0259089 | newly 5 weeks cumul | 2904. | 2669.97 | {2518., 2771.} | 8.05888 | 0.0367982 | newly 53 weeks cumul | 4101. | 4291.8 | {4046., 4483.} | 5.96001 | 0.0251726 |
| 25 | newly 3 weeks spec | 727. | 704.36 | {663., 743.} | 4.18432 | 0.0187223 | newly 3 weeks cumul | 3100. | 2392.8 | {2330., 2448.} | 22.8129 | 0.112547 | newly 5 weeks spec | 309. | 488.45 | {455., 516.} | 58.0744 | 0.198321 | newly 5 weeks cumul | 3847. | 3474.92 | {3395., 3565.} | 9.67195 | 0.0442684 | newly 56 weeks cumul | 4343. | 5301.27 | {5179., 5464.} | 22.0647 | 0.0864933 |
| 26 | newly 3 weeks spec | 523. | 800.69 | {740., 854.} | 53.0956 | 0.184158 | newly 3 weeks cumul | 2145. | 2888.23 | {2740., 3037.} | 34.6494 | 0.128782 | newly 5 weeks spec | 188. | 490.48 | {424., 531.} | 160.894 | 0.414903 | newly 5 weeks cumul | 2653. | 4012.27 | {3740., 4208.} | 51.2352 | 0.179184 | newly 59 weeks cumul | 2904. | 5503.63 | {4916., 5842.} | 89.5189 | 0.27688 |
| 27 | newly 3 weeks spec | 757. | 793.46 | {736., 841.} | 6.27213 | 0.0263509 | newly 3 weeks cumul | 2773. | 2721. | {2562., 2881.} | 3.73963 | 0.0166282 | newly 5 weeks spec | 463. | 533.51 | {495., 570.} | 15.2505 | 0.0609171 | newly 5 weeks cumul | 3879. | 3919.48 | {3736., 4080.} | 3.09771 | 0.0134003 | newly 57 weeks cumul | 5146. | 5781.51 | {5583., 6054.} | 12.4242 | 0.0506604 |
| 28 | newly 3 weeks spec | 747. | 817.42 | {771., 859.} | 9.5261 | 0.0391703 | newly 3 weeks cumul | 3109. | 3064.74 | {2912., 3154.} | 2.2303 | 0.00988045 | newly 5 weeks spec | 347. | 468.37 | {422., 521.} | 34.9769 | 0.128833 | newly 5 weeks cumul | 3954. | 4161.67 | {4016., 4294.} | 5.34117 | 0.0224922 | newly 60 weeks cumul | 4582. | 5420.06 | {5172., 5854.} | 18.2903 | 0.0724637 |
| 29 | newly 3 weeks spec | 724. | 618.56 | {575., 667.} | 14.5635 | 0.0690238 | newly 3 weeks cumul | 2790. | 2071.68 | {1983., 2159.} | 25.7462 | 0.129496 | newly 5 weeks spec | 343. | 449.03 | {407., 493.} | 30.9125 | 0.115821 | newly 5 weeks cumul | 3611. | 3056.48 | {2918., 3188.} | 15.3564 | 0.0726754 | newly 55 weeks cumul | 4378. | 4849.1 | {4545., 5248.} | 10.7606 | 0.0437162 |
| 30 | newly 3 weeks spec | 685. | 626.44 | {577., 673.} | 8.70657 | 0.0402725 | newly 3 weeks cumul | 2400. | 2071.18 | {1983., 2154.} | 13.7008 | 0.0642992 | newly 5 weeks spec | 366. | 467.15 | {424., 512.} | 27.7077 | 0.10506 | newly 5 weeks cumul | 3210. | 3084.57 | {2919., 3202.} | 4.20218 | 0.0189003 | newly 55 weeks cumul | 4078. | 4991.81 | {4513., 5450.} | 22.4083 | 0.0868679 |
| 31 | newly 3 weeks spec | 731. | 767.08 | {700., 840.} | 7.33242 | 0.0305399 | newly 3 weeks cumul | 2627. | 2728.11 | {2570., 2920.} | 5.03959 | 0.0211613 | newly 5 weeks spec | 439. | 485.17 | {419., 558.} | 13.0866 | 0.0522821 | newly 5 weeks cumul | 3631. | 3827.13 | {3578., 4144.} | 6.30322 | 0.0261146 | newly 58 weeks cumul | 4700. | 5388.68 | {4852., 5962.} | 15.2826 | 0.0605842 |
| 32 | newly 3 weeks spec | 583. | 430.27 | {403., 458.} | 26.1973 | 0.132482 | newly 3 weeks cumul | 2074. | 1374.93 | {1301., 1425.} | 33.7064 | 0.178786 | newly 5 weeks spec | 399. | 347.32 | {313., 382.} | 13.183 | 0.0626832 | newly 5 weeks cumul | 2998. | 2110.95 | {1998., 2201.} | 29.5881 | 0.152636 | newly 50 weeks cumul | 4177. | 4110.06 | {3635., 4420.} | 5.22576 | 0.0233316 |
| 33 | newly 3 weeks spec | 525. | 711.71 | {670., 758.} | 35.5638 | 0.131664 | newly 3 weeks cumul | 2485. | 2693.89 | {2610., 2798.} | 8.40604 | 0.0348995 | newly 5 weeks spec | 210. | 421.68 | {393., 449.} | 100.8 | 0.30215 | newly 5 weeks cumul | 3022. | 3674.01 | {3565., 3811.} | 21.5754 | 0.0847 | newly 58 weeks cumul | 3330. | 4945.37 | {4786., 5126.} | 48.5096 | 0.171582 |
| 34 | newly 3 weeks spec | 536. | 790.85 | {728., 840.} | 47.5466 | 0.16823 | newly 3 weeks cumul | 2500. | 2938.42 | {2809., 3044.} | 17.5368 | 0.0699356 | newly 5 weeks spec | 238. | 473.34 | {413., 514.} | 98.8824 | 0.296131 | newly 5 weeks cumul | 3124. | 4032.93 | {3825., 4210.} | 29.0951 | 0.110543 | newly 60 weeks cumul | 3558. | 5422.38 | {4977., 5725.} | 52.3997 | 0.181894 |
| 35 | newly 3 weeks spec | 625. | 602.58 | {545., 640.} | 5.1744 | 0.0234532 | newly 3 weeks cumul | 2116. | 2016.99 | {1816., 2133.} | 5.10444 | 0.02344 | newly 5 weeks spec | 382. | 439.49 | {389., 488.} | 15.4581 | 0.0609103 | newly 5 weeks cumul | 2967. | 2980.62 | {2705., 3130.} | 4.19818 | 0.0184382 | newly 55 weeks cumul | 4257. | 4793.52 | {4434., 5276.} | 12.7263 | 0.0511294 |
| 36 | newly 3 weeks spec | 695. | 569.03 | {522., 615.} | 18.1252 | 0.0876271 | newly 3 weeks cumul | 2481. | 1872.31 | {1788., 1943.} | 24.5341 | 0.122523 | newly 5 weeks spec | 436. | 429.58 | {387., 472.} | 6.16972 | 0.0273545 | newly 5 weeks cumul | 3487. | 2801.54 | {2664., 2935.} | 19.6576 | 0.095406 | newly 53 weeks cumul | 5127. | 4714.83 | {4407., 4977.} | 8.34777 | 0.0382267 |
| 37 | newly 3 weeks spec | 692. | 732.18 | {682., 790.} | 6.77168 | 0.0281454 | newly 3 weeks cumul | 2700. | 2520.98 | {2435., 2628.} | 6.63037 | 0.0299672 | newly 5 weeks spec | 388. | 486.02 | {424., 537.} | 25.2629 | 0.0962302 | newly 5 weeks cumul | 3670. | 3610.94 | {3461., 3786.} | 2.9951 | 0.0131842 | newly 56 weeks cumul | 4686. | 5352.79 | {4866., 5685.} | 14.2294 | 0.0571103 |
| 38 | newly 3 weeks spec | 526. | 878.55 | {829., 919.} | 67.0247 | 0.222435 | newly 3 weeks cumul | 2700. | 3208.51 | {3120., 3303.} | 18.8337 | 0.074832 | newly 5 weeks spec | 147. | 530.7 | {493., 566.} | 261.02 | 0.55699 | newly 5 weeks cumul | 3166. | 4430.05 | {4306., 4540.} | 39.9258 | 0.145801 | newly 59 weeks cumul | 3369. | 6027.53 | {5849., 6158.} | 78.9115 | 0.252541 |
| 39 | newly 3 weeks spec | 662. | 767.51 | {712., 824.} | 16.0438 | 0.0640644 | newly 3 weeks cumul | 2825. | 2804.15 | {2707., 2927.} | 2.61062 | 0.0114389 | newly 5 weeks spec | 284. | 473.92 | {436., 514.} | 66.8732 | 0.221243 | newly 5 weeks cumul | 3546. | 3886.91 | {3749., 4076.} | 9.70699 | 0.039996 | newly 59 weeks cumul | 4001. | 5325.71 | {5146., 5615.} | 33.1095 | 0.123687 |
| 40 | newly 3 weeks spec | 671. | 704.27 | {653., 748.} | 6.19225 | 0.0259811 | newly 3 weeks cumul | 2384. | 2392.35 | {2301., 2490.} | 2.59773 | 0.011375 | newly 5 weeks spec | 421. | 487.43 | {446., 537.} | 15.8409 | 0.0627518 | newly 5 weeks cumul | 3361. | 3469.43 | {3342., 3591.} | 4.17227 | 0.0177893 | newly 56 weeks cumul | 4464. | 5302.42 | {5030., 5553.} | 18.7818 | 0.0743451 |
| 41 | newly 3 weeks spec | 690. | 595.73 | {558., 631.} | 13.6623 | 0.0643007 | newly 3 weeks cumul | 2492. | 2055.86 | {1983., 2127.} | 17.5016 | 0.0837096 | newly 5 weeks spec | 409. | 412.21 | {375., 445.} | 5.6846 | 0.0246349 | newly 5 weeks cumul | 3447. | 2972.39 | {2861., 3058.} | 13.7688 | 0.0644758 | newly 55 weeks cumul | 4485. | 4509.42 | {4280., 4751.} | 3.16299 | 0.0136544 |
| 42 | newly 3 weeks spec | 566. | 440.97 | {406., 470.} | 22.0901 | 0.109098 | newly 3 weeks cumul | 1986. | 1464.97 | {1404., 1518.} | 26.2351 | 0.132374 | newly 5 weeks spec | 394. | 336.64 | {303., 367.} | 14.7259 | 0.0703968 | newly 5 weeks cumul | 2865. | 2189.72 | {2087., 2267.} | 23.57 | 0.116949 | newly 50 weeks cumul | 4248. | 3878.04 | {3631., 4109.} | 9.16714 | 0.0421622 |
| 43 | newly 3 weeks spec | 779. | 924.7 | {866., 974.} | 18.7035 | 0.0740269 | newly 3 weeks cumul | 3579. | 3181.02 | {3049., 3303.} | 11.1199 | 0.0513572 | newly 5 weeks spec | 296. | 589.03 | {533., 631.} | 98.9966 | 0.297956 | newly 5 weeks cumul | 4369. | 4518.76 | {4300., 4670.} | 4.1355 | 0.0175578 | newly 60 weeks cumul | 4830. | 6377.6 | {5998., 6612.} | 32.0414 | 0.120398 |
| 44 | newly 3 weeks spec | 659. | 514.32 | {476., 552.} | 21.9545 | 0.108477 | newly 3 weeks cumul | 2196. | 1646. | {1565., 1711.} | 25.0455 | 0.125599 | newly 5 weeks spec | 414. | 410.06 | {370., 445.} | 5.45894 | 0.0241881 | newly 5 weeks cumul | 3184. | 2521.44 | {2364., 2616.} | 20.809 | 0.10177 | newly 51 weeks cumul | 4755. | 4651.2 | {4162., 4865.} | 3.74175 | 0.0170643 |
| 45 | newly 3 weeks spec | 602. | 660.39 | {607., 701.} | 9.89867 | 0.040391 | newly 3 weeks cumul | 2275. | 2206.3 | {2113., 2297.} | 3.50154 | 0.0156464 | newly 5 weeks spec | 418. | 466.14 | {423., 507.} | 11.9282 | 0.0480028 | newly 5 weeks cumul | 3189. | 3231.91 | {3083., 3395.} | 3.29351 | 0.0141193 | newly 55 weeks cumul | 4496. | 5064.47 | {4749., 5385.} | 12.6439 | 0.0511108 |
| 46 | newly 3 weeks spec | 631. | 554.23 | {505., 604.} | 12.3154 | 0.0578386 | newly 3 weeks cumul | 2297. | 1820.74 | {1725., 1908.} | 20.734 | 0.101299 | newly 5 weeks spec | 464. | 420.56 | {385., 459.} | 9.64655 | 0.0449223 | newly 5 weeks cumul | 3301. | 2735.94 | {2583., 2893.} | 17.1178 | 0.0819464 | newly 53 weeks cumul | 4939. | 4674.15 | {4326., 4985.} | 6.12816 | 0.0278044 |
| 47 | newly 3 weeks spec | 796. | 633.37 | {590., 680.} | 20.4309 | 0.0999171 | newly 3 weeks cumul | 2859. | 2063.63 | {1985., 2144.} | 27.8199 | 0.141773 | newly 5 weeks spec | 468. | 475.23 | {436., 527.} | 5.59188 | 0.0238714 | newly 5 weeks cumul | 3902. | 3089.75 | {2957., 3239.} | 20.8162 | 0.1016 | newly 55 weeks cumul | 5202. | 5105.88 | {4756., 5588.} | 5.18147 | 0.0228156 |
| 48 | newly 3 weeks spec | 746. | 860.72 | {783., 919.} | 15.4611 | 0.061702 | newly 3 weeks cumul | 2942. | 2999.88 | {2783., 3137.} | 4.06186 | 0.0174558 | newly 5 weeks spec | 435. | 560.4 | {501., 612.} | 28.8276 | 0.108711 | newly 5 weeks cumul | 3949. | 4257.41 | {3939., 4485.} | 8.22968 | 0.0340646 | newly 58 weeks cumul | 4973. | 6061.8 | {5517., 6376.} | 21.8942 | 0.0854007 |
| 49 | newly 3 weeks spec | 753. | 880.09 | {842., 913.} | 16.8778 | 0.0674897 | newly 3 weeks cumul | 3170. | 3157.18 | {3092., 3227.} | 1.31356 | 0.00572607 | newly 5 weeks spec | 359. | 541.75 | {513., 570.} | 50.9053 | 0.178259 | newly 5 weeks cumul | 4008. | 4395.59 | {4306., 4480.} | 9.67041 | 0.0400283 | newly 59 weeks cumul | 4662. | 6039.59 | {5918., 6176.} | 29.5493 | 0.112326 |
| 50 | newly 3 weeks spec | 849. | 813.92 | {776., 850.} | 4.48292 | 0.0200888 | newly 3 weeks cumul | 3183. | 2799.57 | {2718., 2860.} | 12.0462 | 0.0558353 | newly 5 weeks spec | 465. | 543.19 | {512., 572.} | 16.8366 | 0.0670433 | newly 5 weeks cumul | 4282. | 4018.94 | {3933., 4087.} | 6.14339 | 0.027585 | newly 57 weeks cumul | 5322. | 5895.03 | {5788., 6012.} | 10.7672 | 0.0443402 |
